# Understanding Digital Technology Access and Use Among New York State Residents to Enhance Dissemination of Health Information

**DOI:** 10.2196/publichealth.4442

**Published:** 2016-04-18

**Authors:** Jennifer A Manganello, Gena Gerstner, Kristen Pergolino, Yvonne Graham, David Strogatz

**Affiliations:** ^1^ Department of Health Policy, Management, and Behavior School of Public Health University at Albany Rensselaer, NY United States; ^2^ New York State Department of Health Office of Minority Health and Health Disparities Prevention Albany, NY United States; ^3^ Department of Epidemiology and Biostatistics School of Public Health University at Albany Rensselaer, NY United States; ^4^ Director, Center for Rural Community Health Bassett Research Institute Cooperstown, NY United States

**Keywords:** media, New York, Internet, health information, eHealth

## Abstract

**Background:**

Many state and local health departments, as well as community organizations, have been using new technologies to disseminate health information to targeted populations. Yet little data exist that show access and use patterns, as well as preferences for receiving health information, at the state level.

**Objective:**

This study was designed to obtain information about media and technology use, and health information seeking patterns, from a sample of New York State (NYS) residents.

**Methods:**

A cross-sectional telephone survey (with mobile phones and landlines) was developed to assess media and technology access, use patterns, and preferences for receiving health information among a sample of 1350 residents in NYS. The survey used random digit dialing methodology. A weighted analysis was conducted utilizing Stata/SE software.

**Results:**

Data suggest that NYS residents have a high level of computer and Internet use; 82% have at least one working computer at home, and 85% use the Internet at least sometimes. Mobile phone use is also high; 90% indicated having a mobile phone, and of those 63% have a smartphone. When asked about preferences for receiving health information from an organization, many people preferred websites (49%); preferences for other sources varied by demographic characteristics.

**Conclusions:**

Findings suggest that the Internet and other technologies are viable ways to reach NYS residents, but agencies and organizations should still consider using traditional methods of communication in some cases, and determine appropriate channels based on the population of interest.

## Introduction

Interventions using digital technologies to improve health, known as eHealth interventions, have become a topic for theoretical discussion and practical application as an effective way to improve or enable health and health care among diverse populations [1-3]. Digital technology, especially the Internet, has become an increasingly popular health intervention tool because of its easy access on a variety of devices (ie, laptops, mobile phones, tablets) and is now a common resource to disseminate and find health information [2,4].

Recent statistics indicate that most people use the Internet [5,6]. In 2013, the Pew Research Center Internet Project Survey reported 85% of adults ages 18 and over are Internet users [5]. However, despite widespread use, variations by age, educational status, and household income remain [6]. National data show that mobile phone use has greatly expanded as well. Recent statistics show that 90% of adults in the United States own a mobile phone, and just more than half (58%) of Americans own a smartphone [7]. Nationally, smartphone ownership is most prevalent among 18-29 year olds (83%) and among those with higher education and income levels [7], which parallels Internet use. The rise in Internet use and smartphone ownership has also led to an increased use of social media (73% of online US adults [8]).

A number of health programs in recent years have used technologies such as text messages and smartphone apps [9,10], and several health interventions have incorporated social media channels [11-13]. Although data on Internet and mobile phone use is widely available at the national level, few states have attempted to collect information at the state level. Many media campaigns and programs that disseminate health information rely on the Internet, mobile phones, and social media channels to provide messages and information at the state level. In order to determine the tools and channels that are most effective in reaching target audiences, it is necessary to understand whether national patterns are representative of media and technology use at the state level and how access and usage may vary among subgroups of the state population.

Working with both internal and external partners, the New York State Department of Health protects the health, productivity, and well-being of all New Yorkers. Of strategic importance is improving the quality and availability of data. Central to this effort is the need for more reliable information about technology use pertaining to the increasingly diverse populations of New York State (NYS). To this end, the New York State Department of Health Office of Minority Health and Health Disparities Prevention (OMH-HDP), in partnership with the University at Albany School of Public Health and Bassett Research Institute, developed the New York State Media and Technology Use Survey study. The aim of the survey was to describe technology use, health information-seeking patterns, and preferences for receiving health information among a sample of NYS residents with oversampling of rural and Hispanic/Latino populations to facilitate future analyses of these subgroups. These subgroups are priority populations to OMH-HDP due to disparate health outcomes and a need to develop and disseminate effective health messages. The analyses for this paper focus on the overall sample of NYS residents; more detailed analyses of the rural and Hispanic/Latino respondents are presented in separate publications [14,15]. In this paper, we address the following questions:

1. What is the level of access to digital technologies, including computers, the Internet, cell phones, smartphones, and texting?
2. What is the frequency of use of various media channels, including email, search engines, online newspapers/magazines, social networking sites (SNS), online videos, video chat, Twitter, online bulletin boards (ie, Pinterest), text messaging, and smartphone apps?
3. What channels are preferred for receiving health information?
4. How do the answers to questions 1 through 3 vary by education, age, sex, ethnicity, race, income, and geographic area?

## Methods

The New York State Media and Technology Use Survey is a cross-sectional telephone survey of a sample of NYS residents, ages 18 years and older. It was created to assess the media and technology access and use of NYS residents, along with health information seeking patterns and preferences. Siena Research Institute (SRI), a public opinion research center that conducts surveys, was hired for data collection. Interviews were conducted via a landline or mobile phone with English and Spanish language options available. Trained interviewers collected data by using the computer assisted telephone interviewing system to conduct telephone interviews. The survey was conducted from August 8 through November 4, 2013, and took about 10 minutes to complete. Institutional review board approval was obtained through the University at Albany Office of Regulatory and Research Compliance. This study was considered exempt from full review.

### Sample

SRI purchased phone number lists generated using a random digit dialing methodology from Survey Sampling International. Random digit dialing was used for the landline sample to ensure selection of both listed and unlisted telephone numbers, whereas the mobile phone sample was retrieved from dedicated wireless telephone exchanges from within NYS. To ensure a sufficient number of rural respondents, a component of the landline sample targeted the 24 NYS counties not situated in a Metropolitan Statistical Area. Oversampling of Hispanic/Latino respondents was accomplished through a similar targeted random sampling of landlines in census tracts with at least a 20% concentration of Hispanic/Latino residents. Some rural and Hispanic/Latino respondents were also identified in the statewide samples of landlines and mobile phones. The sampling plan from these multiple frames produced a study population of 1350 adults, with 483 identified through their mobile phones.

### Measures

The survey asked for demographic information such as geographic area (ie, city/urban, suburban, rural), age, race, ethnicity, sex, education level, employment status, income, number and ages of children, health insurance coverage, and number of doctor visits within the last year. Categories created for race were: White, Black/African American, Asian (ie, Asian Indian, Chinese, Filipino, Japanese, Korean, Vietnamese, Other Asian, Native Hawaiian, Guamanian or Chamorro, Samoan, and Other Pacific Islander), and Other/Multiple. For analyses, Asian categories were combined, and respondents who chose other or multiple categories were compiled into one group. For income, a not sure/missing category was created for respondents who refused to answer or were not sure. For other variables, refusals were changed to missing.

Questions on media and technology access and use included the number of working computers in the home, type of Internet access on these computer(s) (ie, dial up, high speed), whether respondents had cellular service throughout the past year, if they had a plan with unlimited texting, whether the phone was a smartphone, and on what device they typically access the Internet. If respondents reported not having Internet access or a mobile phone, or not using the Internet, a follow-up question was asked to establish the reason. Respondents with Internet access were then asked about Internet use and their frequency of use for a variety of Internet and phone-related activities. All respondents were asked the following: “How often do you do each of the following activities? Do not include times you spend doing these activities as part of your job or school.” A range of activities was provided, such as sending or receiving email, using a search engine, using the Internet to read newspapers/magazines, visiting a SNS site, watching or uploading a video, using video chat, participating on Twitter, using an online bulletin board like Pinterest, receiving or sending a text message on a mobile phone, or using an app on a smartphone. Answer choices were: several times a day, once a day, several times a week, once a week, less than once a week, and never.

The following question was developed to assess respondents’ preferences for receiving health information: “This survey is not providing any health information, but if an organization like the Department of Health wanted to provide health information to people in your community, how would you prefer getting the information? For each way of getting the information, rate your level of interest as low, medium, or high.” The channels of communication to rate were: in-person meeting/workshop, mail to your home (eg, brochure), mobile phone app, text message on a mobile phone, website you could go to, email, social media (ie, Facebook), television, and radio. Channels were rotated in order to avoid any bias related specifically to the order of responses. A transition statement earlier in the survey where questions about health information began stated: “By health information, we mean information about health topics such as exercise, nutrition, immunizations, and where to find a health provider. We do not mean information about the treatment of specific medical conditions.”

### Analysis

Due to the complex sampling strategy, a weighted analysis was conducted utilizing Stata/SE (StataCorp, College Station, Texas). For this sample, weights were derived to adjust for the sampling procedures, which led to some individuals having greater or lesser probability of being included in the survey. A second stage of weighting was used to adjust the distribution of the sample’s socio-demographic characteristics to match the characteristics of the population of NYS residents age 18 and over. Data were weighted for age, sex, region (ie, Upstate New York, Suburban New York City (NYC), NYC-Metro), rural status, race, ethnicity, education, and mobile phone status. Many respondents did not report income; therefore, data were not weighted for income. Chi-square tests were used to compare respondent groups through bivariate (unadjusted) analyses for key demographic variables: education, age, sex, ethnicity, race, income, and geographic area. Ordinal logistic regression and logistic regression were also used to run adjusted models with all demographics accounted for.

## Results

Table 1 presents demographic data for the NYS sample in comparison to all NYS residents. The unweighted sample represents those who participated in the survey, while the weighted sample adjusts for the complex sampling design and also allows for inferences to be made to the general population of the state.

**Table 1:** Key demographics for the sample of New York State respondents (n=1350).

Demographics		NYS Demographics	Unweighted Sample (actual percent surveyed) N (%)	Weighted Sample (weighted estimates)
Education [16]				
	High school grad or less	41%	434 (32)	35%
	Some college/vocational degree	28%	366 (27)	31%
	College graduate or more	31%	537 (40)	35%
Age [17]				
	18-29	22%	289 (22)	23%
	30-49	36%	334 (25)	36%
	50-59	18%	248 (19)	17%
	60 or over	24%	455 (34)	25%
Sex [17]				
	Male	48%	594 (44)	48%
	Female	52%	751 (56)	52%
Ethnicity [18]				
	Hispanic, Latino/a, or Spanish origin	18%	412 (31)	17%
Race [18]				
	White	66%	836 (66)	65%
	Black/African American	16%	162 (13)	16%
	Asian	7%	71 (6)	8%
	Other/Multiple	11%	204 (16)	10%
Household Income [19]				
	Less than $25,000	24%	352 (26)	26%
	$25,000 to $49,999	21%	242 (18)	18%
	$50,000 to $74,999	17%	156 (12)	12%
	$75,000 or more	38%	301 (22)	23%
	Not sure/Missing	N/A	299 (22)	21%
Geographic Area [20]				
	City/Urban	88%	628 (47)	56%
	Suburban	(with urban)	272 (20)	33%
	Rural	12%	435 (33)	11%

**Research Question 1:** What is the level of access to digital technologies, including computers, the Internet, cell phones, smartphones, and texting?

A substantial portion (82%) of the sample reported having at least 1 working computer at home and of those, 1090 answered the follow-up question about whether they have Internet access on home computers; 91% reported having high-speed Internet, and only 19 (2%) respondents answered no.

All respondents were asked about their personal use of the Internet; 85% reported using the Internet at least sometimes. Of those, 53% reported using the Internet several times per day. While using a computer or tablet at home is the main way people reported usually accessing the Internet (62%), 29% indicated they use their cell phones to access the Internet. Respondents with lower education (*P*=.04), or who were younger (*P*<.0001), non-white (*P*<.0001), or nonrural (*P*=.006) were more likely to report using their mobile phone as their main way to access the Internet. A number of respondents (n=221) stated they never use the Internet for personal use (not related to school or work). For these respondents, the most common reasons for not accessing the Internet were: no interest in it (37%), no Internet access (17%), and feel that it is too hard to use (10%).

Overall, mobile phone ownership was very common with 90% of respondents indicating they had a mobile phone. Among the respondents who reported owning a mobile phone (n=1197), 63% had a smartphone, 79% had unlimited texting, and 8% did not have cellular service throughout the entire year.

In response to Research Question 4, computer and mobile phone access were compared by key demographic characteristics. Table 2 shows unadjusted and adjusted comparisons. While there were a number of significant findings in the bivariate analyses, once adjusted, results showed that age was an important predictor across all variables. Older people were less likely to report having home computers, broadband access, mobile phones, smartphones, and unlimited texting. Education and income were also important variables in predicting access to home computers and broadband connections, with higher levels resulting in increased access for both. There were fewer demographic differences for mobile phone and smartphone ownership, with age being the main predictor.

**Research Question 2:** What is the frequency of use of various media channels, including email, search engines, online newspapers/magazines, SNS, online videos, video chat, Twitter, online bulletin boards (ie, Pinterest), text messaging, and smartphone apps?

The most common activities included using email or search engines. Only 21% reported never using email, and only 17% reported never using a search engine. There were a number of activities that a large number of respondents reported never doing: using SNS like Facebook (40%), watching or uploading videos on a site like YouTube (40%), reading newspapers or magazines online (49%), and using video chat services like Skype (61%). Over two-thirds (75%) said they never used SNS for health purposes. Regarding activities specific to mobile phone use, only 17% reported never sending text messages; 26% reported never using mobile phone apps, and over half (56%) said they never used mobile phone apps for health purposes.

To further support findings in response to Research Question 4, Table 3 presents demographic comparisons of activities conducted using the Internet and mobile phones as reported by respondents. A majority of the sample reported never using Twitter (86%) and online bulletin boards like Pinterest (88%); therefore, those 2 activities are not included in the table.

While age was an important predictor of activities involving the Internet and mobile phones, education also appeared to be important, remaining significant even after adjusting for other demographics. Older respondents as well as respondents with lower educational attainment were less likely to report doing most of the activities listed. Some activities—such as online search engine use—could be predicted by income, yet others—such as text messaging—could not. Also of note is that Asian respondents appeared to be much more likely to engage in most activities than other races. Geographic area of residence or being Hispanic/Latino or male (with the exception of using online videos) did not appear to be a significant predictor for any activity after controlling for other demographics.

**Research Question 3:** What channels are preferred for receiving health information?

When asked about receiving health information from an organization, many respondents said they preferred getting information from websites. The general population (49%) rated websites as a high preference, more than any other channel of communication. After websites, the next most preferred channels were television (35%), mail (eg, brochure) (35%), and email (29%). Fewer respondents had a high preference for receiving health information via smartphone apps (25%), in-person meetings (25%), text messages (22%), radio (20%), and SNS (17%).

Table 4 presents comparisons for information preferences across demographics. There are a number of differences by demographics across all methods for receiving health information per the bivariate analyses. When adjusting for all demographics, some notable differences remained. Respondents with higher education were significantly more likely to prefer websites and email, but less likely to prefer TV, than those with lower education levels. Income was also a predictor, with respondents at higher incomes reporting a stronger preference for websites and email.

**Table 2:** Internet and mobile phone use and access for New York State respondents (n=1350 except where noted; weighted estimates, unadjusted and adjusted using logistic regression^1^).

		% working computer at home	% broadband access at home (n=1093)	% have mobile phone	% have unlimited texting (n=1197)	% have smartphone (n=1197)
Education		*P*<.0001	*P*=.0003	*P*=.0005	*P*=.0779	*P*=.0006
	High school graduate or less	66%	88%	85%	74%	54%
	Some college/vocational degree	84%**	93%	89%	82%	64%*
	College graduate or more	96%***	97%*	95%	80%	70%**
						
Age		*P*<.0001	*P*<.0001	*P*<.0001	*P*<.0001	*P*<.0001
	18-29	89%	97%	93%	90%	86%
	30-49	88%	96%	94%	85%	73%**
	50-59	82%*	92%**	96%	75%**	51%***
	60 or over	66%***	85%***	75%***	55%***	28%***
						
Sex		*P*=.0482	*P*=.6778	*P*=.0030	*P*=.1003	*P*=.0147
	Female	79%	93%	87%	76%	58%
	Male	85%	93%	93%	81%	67%
						
Ethnicity		*P*=.0441	*P*=.2314	*P*=.0002	*P*=.6391	*P*=.0008
	Non-Hispanic, Latino/a, or Spanish origin	83%	93%	91%	79%	61%
	Hispanic, Latino/a, or Spanish origin	78%	91%*	83%*	80%	73%
						
Race		*P*<.0001	*P*=.6919	*P*=.4203	*P*=.0157	*P*<.0001
	White	83%	93%	90%	75%	56%
	Black/African American	73%	90%	90%	89%*	73%**
	Asian	97%***	94%	91%	80%	93%**
	Other/Multiple	77%	94%	84%	83%	63%
						
Household Income		*P*<.0001	*P*<.0001	*P*<.0001	*P*=.1523	*P*=.0110
	Less than $25,000	68%	90%	86%	82%	60%
	$25,000 to $49,999	85%**	88%	89%	80%	63%
	$50,000 to $74,999	92%**	97%	97%*	77%	59%
	$75,000 or more	97%***	99%**	96%*	80%	73%**
	Not sure/Missing	73%	89%	83%	71%	55%
						
Geographic Area		*P*=.0006	*P*=.0018	*P*=.0007	*P*=.0235	*P*=.0003
	Urban	79%	94%	90%	80%	66%
	Suburban	89%	95%	93%	80%	64%
	Rural	75%	84%*	79%**	67%	43%

Reference group is the first group listed for each demographic characteristic.

^1^Unadjusted P-values of significance are indicated in the table.

Adjusted P-values of significance are noted as follows: *<.05; **<.01; ***<.0001

**Table 3:** Frequency of Internet- and mobile phone-related activities for New York State respondents (n=1350; weighted estimates, unadjusted and adjusted using ordinal logistic regression^1^).

		Internet use: % Never	Email: % Never	Search engine: % Never	Read news/mags: % Never	SNS: % Never	SNS for health: % Never	Use videos: % Never	Video chat: % Never	Texts: % Never	Apps: % Never	Health apps: % Never
Education		*P*<.0001	*P*<.0001	*P*<.0001	*P*<.0001	*P*<.0001	*P*=.0108	*P*<.0001	*P*<.0001	*P*<.0001	*P*<.0001	*P*=.8264
	High school or less	30%	40%	34%	70%	53%	69%	55%	77%	25%	43%	55%
	Some college/Vocational degree	12%***	17%***	14%***	46%***	34%**	73%	33%**	59%**	15%***	23%**	52%
	College graduate or more	3%***	6%***	3%***	29%***	31%**	82%	27%**	47%***	10%**	13%***	58%
												
Age		*P*<.0001	*P*<.0001	*P*<.0001	*P*<.0001	*P*<.0001	*P*=.2962	*P*<.0001	*P*<.0001	*P*<.0001	*P*<.0001	*P*=.0062
	18-29	4%	10%	5%	37%	14%	72%	15%	50%	1%	9%	47%
	30-49	7%***	13%	9%***	40%	28%***	78%	28%***	54%***	8%***	19%***	53%
	50-59	16%***	25%***	19%***	55%***	54%***	76%	45%***	72%***	18%***	28%***	74%**
	60 or over	35%***	40%***	38%***	67%***	69%***	76%	71%***	82%***	48%***	66%***	69%*
												
Sex		*P*=.2818	*P*=.0361	*P*=.0080	*P*=.0002	*P*=.4823	*P*=.3305	*P*=.0001	*P*=.0223	*P*=.0808	*P*=.0644	*P*=.2048
	Female	17%	24%	20%	53%	39%	74%	42%	61%	19%	30%	50%
	Male	12%	17%	14%	44%**	40%**	77%	35%*	61%	14%	21%	60%
												
Hispanic, Latino/a, or Spanish		*P*=.3955	*P*=.0282	*P*=.2321	*P*=.3031	*P*=.1661	*P*=.0001	*P*=.0087	*P*=.0264	*P*=.0536	*P*=.0530	*P*=.5566
	No	14%	20%	16%	48%	40%	79%	39%	60%	17%	25%	56%
	Yes	16%	25%	19%	51%	34%	61%	35%	62%	11%	28%	51%
												
Race		*P*=.0001	*P*=.0006	*P*<.0001	*P*=.0026	*P*=.0059	*P*=.1144	*P*<.0001	*P*<.0001	*P*=.0061	*P*=.0362	*P*=.8826
	White	14%	20%	16%	48%	43%	79%	41%	62%	19%	25%	58%
	Black/African American	23%*	28%	25%	56%	39%	76%	42%	67%	14%	30%	53%
	Asian	3%	5%	4%*	27%*	21%	65%	20%*	34%*	4%	16%	48%
	Other/Multiple	17%	26%	20%	49%	33%	64%	34%	59%	12%	30%	49%
												
Household Income		*P*<.0001	*P*<.0001	*P*<.0001	*P*<.0001	*P*=.7770	*P*=.0002	*P*<.0001	*P*=.0015	*P*=.0725	*P*<.0001	*P*=.0210
	Less than $25,0000	23%	31%	28%	57%	41%	60%	49%	65%	17%	34%	59%
	$25,000 to $49,999	14%	20%	13%**	53%	41%	72%	40%	67%	17%	34%	51%
	$50,000 to $74,999	5%*	9%*	4%***	31%	31%	90%***	24%	50%	16%	11%**	55%
	$75,000 or more	2%***	7%***	5%***	57%**	37%	81%*	26%*	51%	12%	9%**	54%
	Not sure/Missing	26%	33%	31%	34%	45%	79%**	49%	69%	23%	37%	72%**
												
Geographic Area		*P*=.0004	*P*=.0001	*P*=.0002	*P*=.0788	*P*=.0194	*P*=.1711	*P*=.0032	*P*=.0291	*P*=.0003	*P*=.0049	*P*=.7291
	Urban	16%	23%	19%	48%	38%	71%	39%	60%	16%	26%	55%
	Suburban	9%	14%	11%	47%	39%	82%	34%	58%	15%	19%	55%
	Rural	20%	29%	22%	57%	50%	77%	52%	75%	25%	45%	61%

Reference group is the first group listed for each demographic characteristic.

^1^Unadjusted P-values of significance are indicated in the table.

Adjusted P-values of significance are noted as follows: *<.05; **<.01; ***<.0001

**Table 4:** Preferred media channels for receiving health information for New York State respondents (n=1350; weighted estimates, unadjusted and adjusted using ordinal logistic regression^1^).

		Website: % High	TV: % High	Mail: %High	Email: % High	In-person: % High	Phone app: % High	Text: % High	Radio: % High	SNS: % High
Education		*P*<.0001	*P*=.0044	*P*=.4485	*P*<.0001	*P*=.0884	*P*=.0333	*P*=.0844	*P*=.8113	*P*=.1356
	High school grad or less	33%	43%	35%	24%	25%	21%	26%	21%	19%
	Some college/vocational degree	53%***	32%*	38%	29%*	29%	25%*	22%	20%	15%
	College graduate or more	62%***	29%**	32%	33%**	22%	27%	16%*	17%	15%
										
Age		*P*<.0001	*P*=.7332	*P*=.0851	*P*<.0001	*P*=.0120	*P*<.0001	*P*<.0001	*P*=.3081	*P*<.0001
	18-29	62%	33%	30%	33%	26%	37%	31%	19%	28%
	30-49	55%***	33%	32%	35%	24%	32%*	29%	19%	20%**
	50-59	51%***	39%*	37%*	27%*	32%	15%***	14%***	26%	12%***
	60 or over	28%***	36%	43%**	16%***	21%	9%***	7%***	17%	5%***
										
Sex		*P*=.2074	*P*=.1689	*P*=.0025	*P*=.6626	*P*=.8212	*P*=.1646	*P*=.9592	*P*=.4376	*P*=.6992
	Female	47%	36%	41%	27%	26%	24%	21%	19%	18%
	Male	51%	33%*	29%*	30%	25%	26%	22%	21%	16%*
										
Hispanic, Latino/a, or Spanish		*P*=.8131	*P*=.0002	*P*=.0002	*P*=.0083	*P*<.0001	*P*<.0001	*P*<.0001	*P*=.0949	*P*<.0001
	No	49%	33%	34%	27%	24%	23%	19%	19%	15%
	Yes	48%	44%*	44%**	36%	31%	36%*	32%	23%	27%*
										
Race		*P*=.0001	*P*=.0350	*P*=.0179	*P*=.0068	*P*=.0749	*P*<.0001	*P*<.0001	*P*=.0013	*P*<.0001
	White	49%	31%	34%	25%	23%	19%	15%	17%	11%
	Black/African American	55%*	42%*	41%	36%	30%	33%**	35%**	30%	23%*
	Asian	41%	35%	18%	31%	27%	37%*	21%	9%	26%**
	Other/Multiple	47%	47%*	44%	40%*	32%*	37%*	38%*	30%*	32%*
										
Household Income		*P*<.0001	*P*=.0073	*P*=.0359	*P*<.0001	*P*=.3229	*P*=.0019	*P*=.003	*P*=.3551	*P*=.1207
	Less than $25,0000	38%	40%	39%	27%	26%	22%	27%	22%	22%
	$25,000 to $49,999	52%	40%	30%	27%	24%	26%	23%	18%	14%*
	$50,000 to $74,999	56%*	32%	30%*	27%	23%	26%	12%**	20%	19%
	$75,000 or more	68%***	28%	31%*	40%***	22%	33%**	21%	21%	14%
	Not sure/Missing	34%	33%	34%**	21%	26%	16%	20%*	19%*	14%
										
Geographic area		*P*=.0178	*P*=.4264	*P*=.2260	*P*=.0167	*P*=.2434	*P*=.0011	*P*<.0001	*P*=.0582	*P*=.0040
	Urban	49%	36%	32%	32%	27%	28%	27%	22%	20%
	Suburban	53%	34%	38%*	27%	25%	23%	16%	17%	14%
	Rural	42%	32%	39%	20%	17%	13%	11%**	14%	10%

Reference group is the first group listed for each demographic characteristic.

^1^Unadjusted P-values of significance are indicated in the table.

Adjusted P-values of significance are noted as follows: *<.05; **<.01; ***<.0001

## Discussion

### Principal Findings

For this sample, there was high Internet and mobile phone use, and many respondents had high-speed Internet access. This group also reported a high level of engagement with Internet- and mobile phone-related activities, especially with texting. Thus, NYS respondents are similar in many ways to national samples regarding Internet use and mobile phone ownership. In fact, some percentages are identical; 85% of NYS respondents in this sample report using the Internet, compared to 85% nationally, and 90% of respondents have a mobile phone, compared to 90% nationally [5,21]. Considering these high numbers, it appears reasonable that organizations can use the Internet and mobile phones to disseminate health information and support interventions.

Still, there are some differences across demographics. Similar to national data noted in the Introduction section, this research found that those who are older, have fewer years of education, and are in lower income brackets are less likely to use the Internet. Age and income, and sometimes education, also seem to be important in explaining differences in usage of technologies and preferences for information. These results are similar to what has been found with national samples [22]. While national data show similar trends for smartphone use, income and education do not seem to impact smartphone use as much as age does in NYS. And, different from other research [23], in this sample, income was the biggest predictor of using SNS for health, as opposed to age; those with lower incomes were more likely to use SNS for health.

Despite the widespread access to digital technologies, these findings suggest there are many variations in what people *do* online and with their mobile phones. For instance, respondents were more likely to use text messages than SNS and, when compared to national results of the 2013 Pew Research Internet Project (73%), this sample used SNS less (60%) (SNS use for health was similar to that in other research, 25% compared to 32% [23]). It is also important to remember there are variations not measured here with respect to specific websites or tools. Different groups may prefer different SNS. For example, LinkedIn users tend to be highly educated and come from higher income households [24].

Given the variation among Internet and mobile phone activities, it is recommended that public health groups seeking to disseminate health information should consider specific technology access and use patterns and preferences of the target population when developing a communication plan. Although there are a number of new and unique channels, many NYS respondents preferred getting information from organizations on websites. Having a website where people can go for information is a useful strategy, with other channels such as text messages, social media, and videos used to not only publicize the website but to also provide alternative modes of communication. It is of interest that television and mail (ie, a brochure) were the second and third preferred modes of communication, showing that even with the increased utilization of digital technologies, traditional communication channels are still viable methods for sharing information. Of note, those who were older or reported less education were less likely to prefer websites and email.

Even though these data support the idea that digital channels for communication can effectively and efficiently reach many people, they may not be the only channels to use when disseminating health-related resources and information, especially for certain populations. Traditional information sources such as brochures, television, billboards, bus signs, and radio should be considered as well. Offline information sources could be developed to supplement any online information dissemination activities. This would allow better access for those who do not regularly interact with online or new technology platforms.

Further, the goals of any public health communication program or intervention should be evaluated using data to inform decisions about which information channels would be most effective at reaching the target population. It is also important to consider the pros and cons of each channel being considered. For instance, while many use social media to deliver information, the strength of social media is that people can interact with each other and provide user-generated comments and information. There are instances where this may be ideal, but this approach could also lead to issues such as misinformation.

### Limitations

While this study provides important information from a sample of NYS residents, there were many complexities associated with the nature of a cross-sectional phone survey, compounded by the ever-changing dynamics of media and technology. Among them, there was limited time to conduct the survey. Questions were tested prior to finalizing the survey to ensure that the call lasted only 10 minutes. Although a substantial number of questions were asked, we did not have time to fully explore respondents’ experiences using media and technology for health information. Sample selection was intended to target specific subpopulations (ie, Rural, Hispanic/Latino, mobile phone users). To ensure that the sample was representative of NYS, weighted adjustments were calculated to provide a more accurate depiction. In addition, interviewers did not ask about all possible communication channels. While questions referred to social media sites, respondents may not have considered sites like YouTube or blogs as belonging in that category. These channels could prove useful depending on the target population, and video-based sites like YouTube might be especially suitable to some groups. Finally, we asked about preferences for receiving information in a general way. It may be that responses could have been different if asked about specific health topics. However, we did provide guidance as to what we meant by “health information.” It may also be that respondents interpreted “if an organization like the Department of Health” in different ways; people may not be familiar with the work of the Department of Health, or think it only has certain functions, such as tracking diseases. This may have impacted responses to this question.

### Conclusions

This study represents an important first step in exploring media and technology use for public health purposes at the state level, but there is a need for future research on specific populations to examine how variables beyond the demographics presented here impact media and technology use, and health information-seeking activities and preferences. Factors such as language, culture, and health literacy could all potentially impact media and technology use and preferences for receiving health information. For example, there is a growing realization that even though there has been a decrease in the digital divide with respect to access, there is a growing divide regarding skills [25]. Additional research can also explore how preferences for information dissemination may vary by topic.

While there are limitations that must be considered when drawing conclusions from this study, the data collected suggests that people in NYS are online and engaged in technology use but may have unique preferences for receiving health information. When considering how to disseminate health information, it is important to ensure that the methods being used are appropriate for the target population. Technology is constantly evolving and trends are always changing. A SNS that may be popular today may be displaced by a new one in the future. Continued efforts to understand and stay abreast of technology use patterns and preferences for receiving health information will be an important goal as we consider ways to use digital technologies to improve public health.

## Abbreviations

NYC: New York City
NYS: New York State
OMH-HDP: Office of Minority Health and Health Disparities Prevention
SNS: social networking sites
SRI: Siena Research Institute
